# Irish *Cepaea nemoralis* Land Snails Have a Cryptic Franco-Iberian Origin That Is Most Easily Explained by the Movements of Mesolithic Humans

**DOI:** 10.1371/journal.pone.0065792

**Published:** 2013-06-19

**Authors:** Adele J. Grindon, Angus Davison

**Affiliations:** School of Biology, University of Nottingham, Nottingham, Nottinghamshire, United Kingdom; Institut de Biologia Evolutiva - Universitat Pompeu Fabra, Spain

## Abstract

The origins of flora and fauna that are only found in Ireland and Iberia, but which are absent from intervening countries, is one of the enduring questions of biogeography. As Southern French, Iberian and Irish populations of the land snail *Cepaea nemoralis* sometimes have a similar shell character, we used mitochondrial phylogenies to begin to understand if there is a shared “Lusitanian” history. Although much of Europe contains snails with A and D lineages, by far the majority of Irish individuals have a lineage, C, that in mainland Europe was only found in a restricted region of the Eastern Pyrenees. A past extinction of lineage C in the rest of Europe cannot be ruled out, but as there is a more than 8000 year continuous record of *Cepaea* fossils in Ireland, the species has long been a food source in the Pyrenees, and the Garonne river that flanks the Pyrenees is an ancient human route to the Atlantic, then we suggest that the unusual distribution of the C lineage is most easily explained by the movements of Mesolithic humans. If other Irish species have a similarly cryptic Lusitanian element, then this raises the possibility of a more widespread and significant pattern.

## Introduction

The geographic origins of the flora and fauna of Ireland are mysterious because a number of species, including the strawberry tree, the Kerry slug, and the Pyrenean glass snail, are exclusive to Ireland and Iberia. In 1846 Forbes described this general pattern as ‘Lusitanian’, which subsequently was called, perhaps with some irony, ‘the Irish question’ [1]. To date, the origins of the Ireland-Iberia connection remain unclear, with no single theory able to explain every pattern. More recently, genetic studies have made great progress, because it has been possible to finely dissect past events on a case-by-case basis [2], [3]. Moreover, a few more detailed studies have begun to show that patterns of variation are not always concordant between different genetic markers, because of different histories and selection [4], [5], [6]. Of particular note, new inferences from small mammal phylogeography have led to the proposal that the characteristic signature seen in some Irish species and the “Celtic fringe” in general is as a result of insular isolation – a first wave of colonisation across Britain and Ireland was later followed by second wave that only reached mainland Britain, but not the western fringes and certainly not Ireland [4].

With a view to further understanding the intriguing affinities of the Irish fauna, we chose to study the widespread and common European land snail species, *Cepaea nemoralis*. Fossil material indicates that this species has been continuously present in Ireland for at least 8000 years (Newlands Cross, Co. Dublin: 7600+/−500 BP Cartronmacmanus, Co. Mayo: 8207+/−165) [7], [8]. On the West coast, the normally rare white-lipped shell form is common and populations are also much larger than average. As one of the few other regions known to contain populations with both white-lipped and large shelled individuals is in the Pyrenees and Cantabria, Cook & Peake [9] speculated that the some Irish *C. nemoralis* might originate from this region. Moore [1] went so far as to suggest that Irish snails might have been brought across the sea by Mesolithic humans. However, convergent evolution through natural selection might also explain the similarity, especially since some areas of Western Ireland and Northern Spain have a similar underlying geology.

As a preliminary mitochondrial DNA study showed that two populations in Ireland were genetically distinct from those in Britain [10], we thought it conceivable that Irish *C. nemoralis* might originate, at least in part, from a Southern French or Iberian source. We therefore set out to establish the post-glacial geographic origin of Irish *C. nemoralis*, by sampling widely across the whole of Europe, with a particular concentration of sites in Ireland, Britain, and across Northern Spain and Southern France, including the Pyrenees. In order to achieve this, a fragment of the mitochondrial genes cytochrome oxidase subunit I (COI) and 16S rRNA were used to construct phylogenies. The results may have implications for our understanding of the human colonisation of Ireland.

## Materials and Methods

### Ethics statement

All animal work was conducted according to relevant national and international guidelines. No specific permissions are required to work with invertebrates in the UK. Similarly, no specific permissions were required for the collection of snails from sample sites, because they were not collected from protected areas of land. The land snail, *C. nemoralis*, is not an endangered or protected species.

### Samples

*Cepaea nemoralis* is widespread across Western Europe, reaching its northern limit at roughly 60°N and stretching as far south as approximately 39°N [11]. In Iberia, it is only found along the Northern coast and on adjacent coastal mountain ranges, with some doubt as to whether Portuguese populations are a recent introduction. It is not found in the interior of Spain, nor the Mediterranean coast to the East of the Pyrenees. The shell is generally globular in appearance and slightly depressed, ranging in size from 12 by 18 mm to 26 by 28 mm, with the largest shelled individuals being found in the Pyrenees, Italy, and on the West coast of Ireland. In a few European locations, including sites in Cantabria in Spain, the Pyrenees, Denmark, Yorkshire and Cornwall in England, North Wales, Western Scotland; and the West coast of Ireland, the normally dark lip colour is instead polymorphic, being white/brown/black .

Samples were obtained between 2005 and 2007 by volunteer-led collection and by field trips to the more remote or important locations in France, Spain and the Pyrenees. Populations were located by thorough inspection of suitable habitats, with care taken so as not to sample close to sites where recent introductions are likely, such as agricultural areas, parks, or private gardens. Where possible, between 10 and 30 individuals were collected at each site, from an area no larger than 10×10 metres. We were also able to make use of the British samples (36 sites, 423 individuals) and sequences reported in Davison [10].

### DNA extraction, amplification, and sequencing

Thin slices of foot muscle were removed using a scalpel and DNA was extracted using the protocol of Doyle and Doyle [12]. A ∼600 base pair (b.p.) fragment of the mitochondrial gene COI was amplified by the polymerase chain reaction (PCR), using either the Folmer *et al.* [13] primers, those of Gittenberger et al. [14], or a combination of the two. PCR conditions for Folmer primers were as described, using an annealing temperature of 55°C, but for the Gittenberger primers, or a combination of both sets, the PCR conditions used an annealing temperature of 45°C. Additionally, a 420 bp fragment of the mitochondrial gene 16S rRNA was amplified using the primers 16sar (5′-CGCCTGTTTATCAAAAACAT-3′) and 16sbr (5′-CCGGTCTGAACTCTGATCAT-3′). A nuclear gene ITS was also amplified using the primers LSU-1 (5′-CTAGCTGCGAGAATTAATGTGA-3′) and LSU-3 (5′-ACTTTCCCTCACGGTACTTG-3′), but almost lacked variation and so was not used further. Subsequently, the PCR product was sequenced with forward primers (and reverse, if necessary) using BigDye™ Terminator version 3.0 Cycle Sequencing Kit

### Analyses

Sequences were aligned by eye in Bioedit version 7.0.9 [15], and subsequently collapsed manually into individual haplotypes. To visually check for substitution saturation, the number of transitions and transversions was plotted against genetic distance (total sequence divergence for all pairwise comparisons of the data). In addition, an entropy-based statistical test was used which takes into account tree topology, sequence length, and the number of taxa [16]. In this test, if Iss (i.e. index of substitution saturation) is smaller than Iss.c (i.e. critical Iss), then it is concluded that the sequences have not experienced substitution saturation. Both tests were implemented in DAMBE version 5.0.19 [17]. Sequences were also tested for evidence of recombination, using Recco [18].

To infer the model of nucleotide substitution that best fits the dataset, the Akaike Information Criterion (AIC) and Bayesian methods (i.e. BIC) were used because they simultaneously compare multiple nested or non-nested models, assess the uncertainty of a model, as well as estimating phylogenies and model parameters using all the known models [19]. The model of DNA evolution was established using ModelTest version 3.7 [20], jModelTest 0.1.1 [21], and Kakusan3 [22], taking into consideration the AIC or BIC test results. Phylogenies with the appropriate model of evolution were then constructed using the following methods: neighbour-joining (NJ – PAUP* 4b10) [23], maximum likelihood (ML – Phyml v2.4.4) [24], approximate Likelihood Ratio Tests (aLRTs – Phylml v2.4.4) [25], and Bayesian analysis MrBayes v3.1 [26]. Note that as the rate of mitochondrial evolution is completely uncertain for *Cepaea* mtDNA, we did not attempt to use a molecular clock - inappropriate calibration may have misled substantial numbers of phylogeographic studies [27].

The criteria used for NJ trees were: rate matrix, base frequency, shape parameter of the gamma distribution (based on 16 rate categories), and proportion of invariant sites all estimated using maximum likelihood inferred from an initial NJ phylogeny. The parameters calculated from the initial tree were then used to construct a new NJ tree with the parameters being estimated again. This procedure was repeated until the likelihood value was stable. In addition, bootstrap values were calculated using 1000 replicates to determine the support for branches within the NJ tree. ML trees were constructed using PhyML, both the gamma shape parameter and proportion of invariant sites were estimated from the data. ML bootstrap values were also calculated using 1000 replicates. Finally, Bayesian inference was performed using MrBayes, by means of a 4 chain Markov Chain Monte Carlo algorithm for an initial 500000 generations, sampling every 100 generations, with a subsequent 500000 generations until the diagnostic value was <0.01, indicating that convergence had occurred. The heating parameter was set to 0.2 and a ‘burnin’ value of 1250, with the estimated variables being calculated using three distance states.

The relationship between haplotypes of the same lineage was visualised using median-joining (MJ) networks, drawn in Network version 4.5.0.7 [28]. To ensure an accurate representation of the relationship between haplotypes, the MJ networks were calculated and cleaned up using the Maximum-Parsimony (MP) option, which removes any unnecessary median-vectors and links.

The genetic structure between populations was estimated by calculating population pairwise estimates of F*_ST_* in Arlequin version 3.0 [29]. The haplotypes are permuted between populations in order to calculate the null distribution of pairwise F*_ST_* values under the hypothesis of no differentiation between populations. The *P* value is the proportion of permutations that lead to a F*_ST_* value which is larger or equal to the observed one. Regression plots of F*_ST_* values against ln [distance] were constructed to test for an association of geographical distance with genetic distance, and the significance determined by a Mantel test in Arlequin [29]. Also implemented in Arlequin, the nucleotide and haplotype diversities were calculated for populations with five or more individuals.

## Results

Cytochrome oxidase sequences (627 b.p.) were obtained from 880 individuals from 111 European populations. Subsequently, these sequences were collapsed into 340 unique haplotypes. Recco did not infer recombination, and no evidence of saturation was observed for the sequences. The GTR+I+G model of nucleotide substitution was selected by the AIC method in ModelTest, jModeltest, and Kakusan.

**Figure 1:**
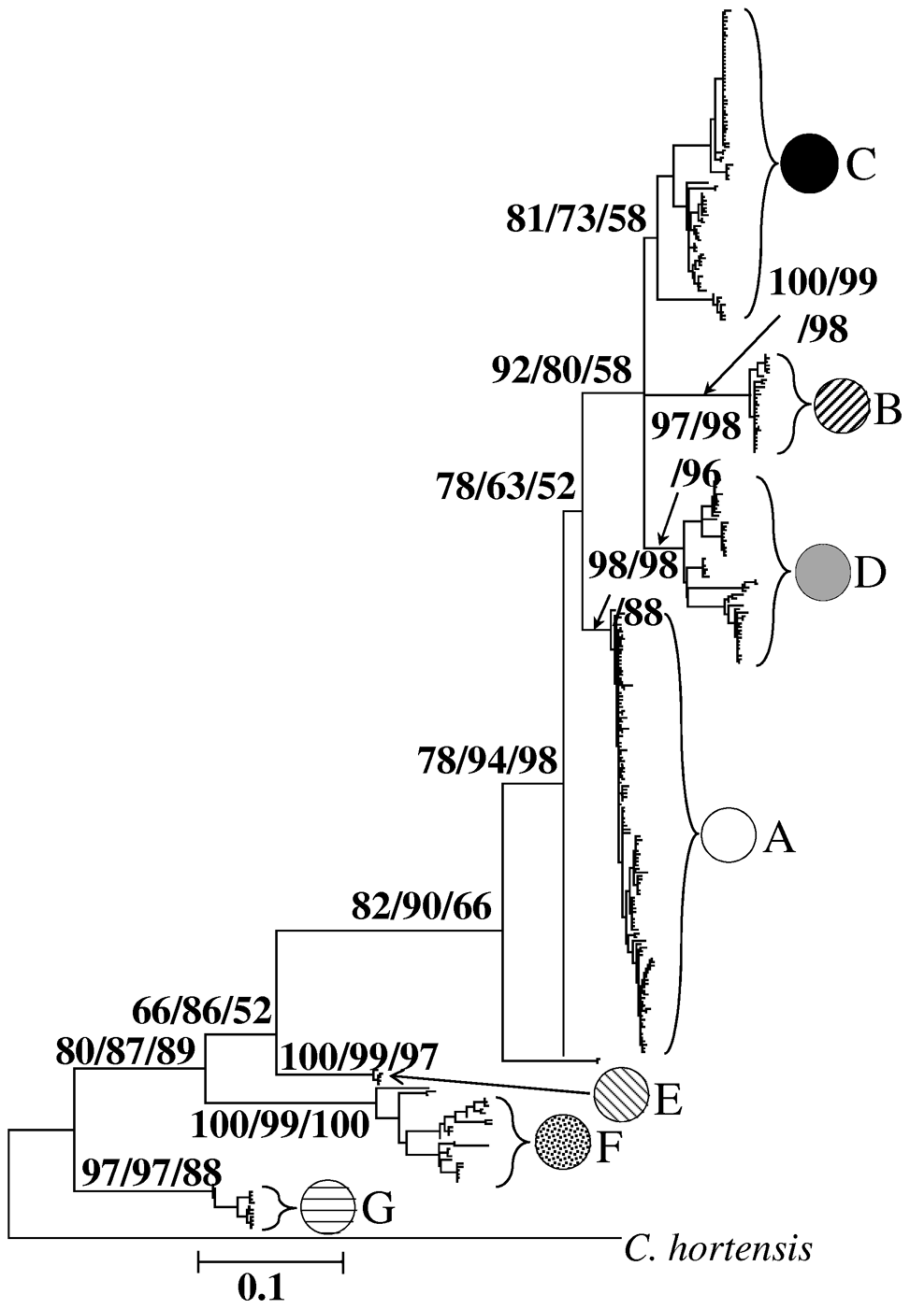
Phylogeny of *C.* nemoralis cytochrome oxidase haplotypes. Support values >50% are displayed on main branches, obtained using neighbour-joining, maximum likelihood and Bayesian methods respectively. Pie charts refer to the same lineages as Figure 2.

**Figure 2:**
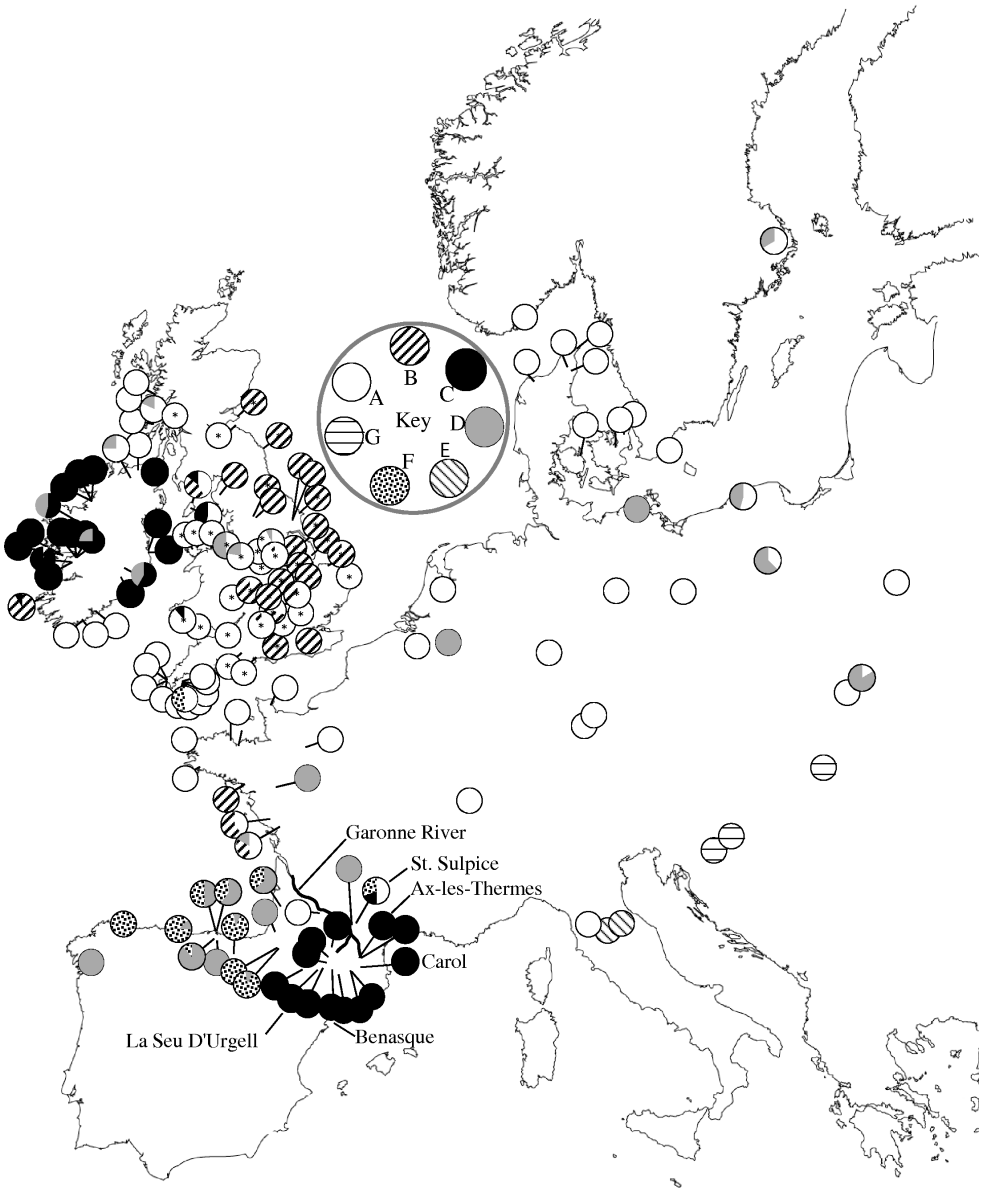
Distribution of main *C. nemoralis* mitochondrial lineages across Europe. Some samples that have been used previously [10], all British, are indicated by an * in the pie chart. Pyrenean locations that share identical haplotypes with Ireland are shown.

Phylogenetic methods placed both the COI (Figure 1) and 16S rRNA haplotypes (not shown) into 7 well supported main lineages (A to G), with all except B being found in one of the three classic refugial areas, Southern France and Iberia (A, C, D, F), Italy (A, E) or the Balkans (G) (Supplementary Tables S1, S2; Figure 2; Genbank accession numbers KC954776 - KC955122 and KC967128-KC967199).

The majority of Europe contains snails with haplotypes belonging to the A lineage, but lineage D is also widespread, being found predominantly in Spain and Ireland, but also common in Sweden, France, Germany and Poland. In the UK, five lineages were found, A, B, C, D, and F, though only two were common (A, B), having an East-West distribution (Figure 2) [10]. Lineage C was only found in one snail from Wales; lineage F was only found in two snails in the extreme West, in Cornwall; lineage D was only found in two snails in West Scotland. Within Iberia, each lineage is geographically restricted: lineage C haplotypes were only found in the central and Eastern Pyrenees; lineage D and F haplotypes were found in the Western Pyrenees and the North West coast. The snails of Ireland are relatively diverse – four lineages were found in Ireland (A, B, C, D), with by far the majority of sampled Irish individuals (73%) having a mitochondrial DNA lineage (C) that was only elsewhere found in the Central (Andorran) and Eastern Pyrenean region, as well as St Sulpice, the Isle of Man and a single specimen from Wales.

**Figure 3:**
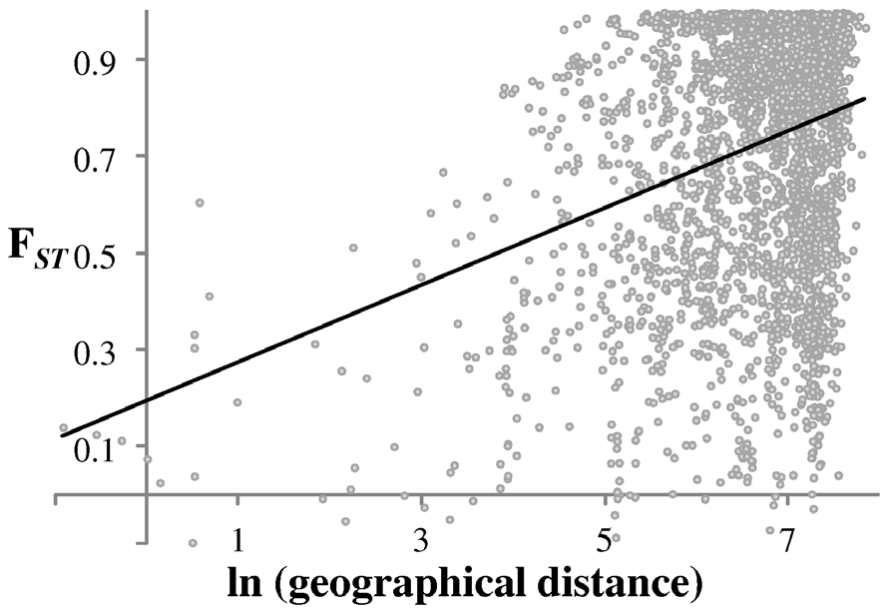
Linear regression of genetic versus geographic distance. F*_ST_* against geographical distance for *C. nemoralis* populations (*b* = 0.0791; *P*<0.0001).

**Figure 4:**
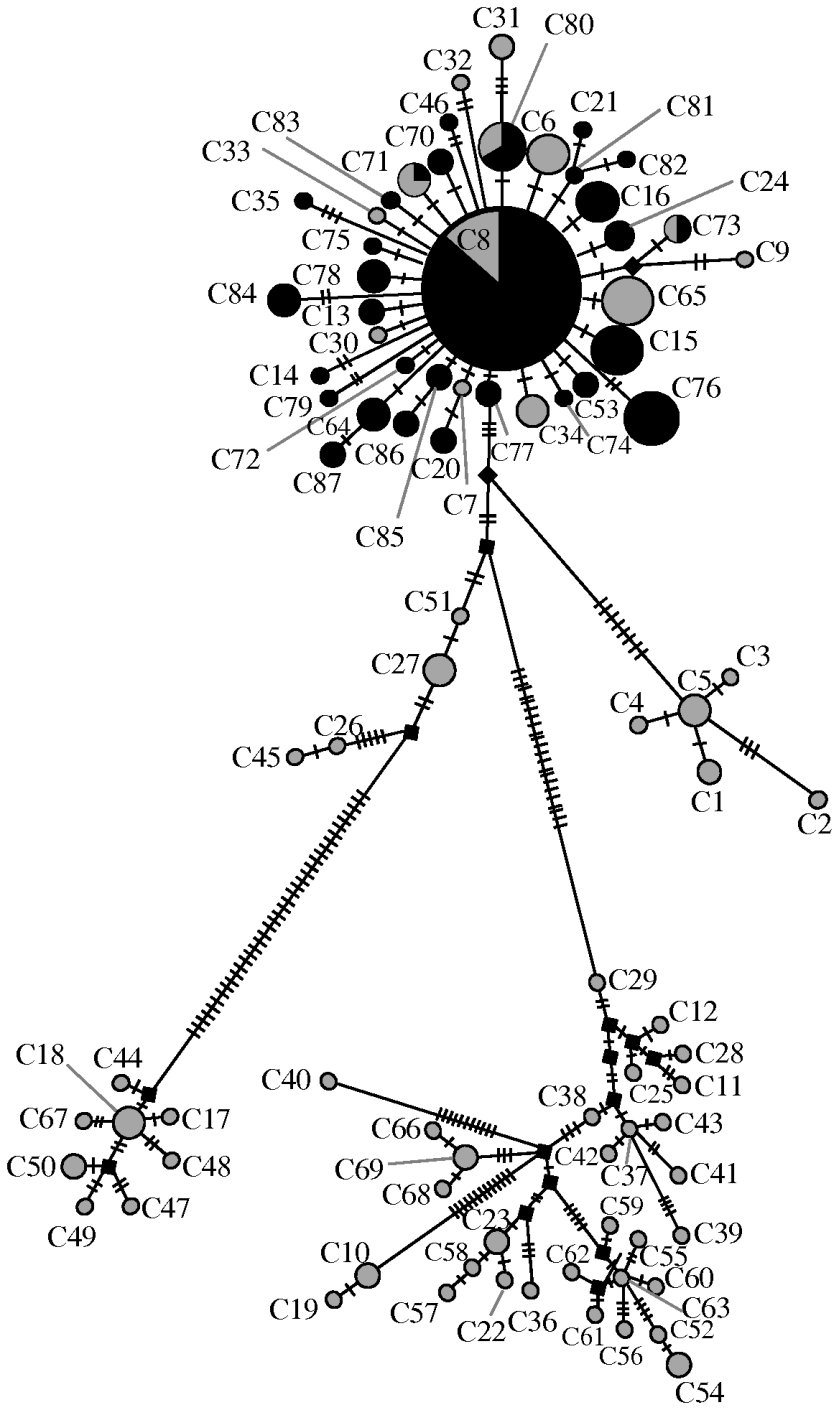
Median-joining network showing the relationship between haplotypes of lineage C. Size of the circle is proportional to the number sampled. Dashes indicate hypothesised but unsampled haplotypes. Shading represents the location of individual haplotypes, black for Ireland and the Isle of Man, grey for Spain and France.

The majority of pairwise comparisons of F*_ST_* were high and significantly different from zero (mean = 0.72; Supplementary Table S3). There was also a significant association of F*_ST_* with geographical distance (*b* = 0.0791; *P*<0.0001; Figure 3). However, ten comparisons between geographically distant Irish and Pyrenean populations were non-significantly different from zero (Table 1). This is because four haplotypes (C8, C71, C73, C80) were shared between Ireland and Carol/Ax Les Thermes in France, and, Ireland and La Seu D'Urgell/Benasque in Spain. The median joining network of lineage C (Figure 4) shows that C8, the most common haplotype with lineage C, is at the centre of a star-like radiation. Both C71 and C80 differ from C8 by a single substitution.

**Table 1:** F*_ST_* comparisons between Irish and Pyrenean populations that are non-significantly different from zero.

Ireland	Pyrenees
Doolin	Carol
Graffy	La Seu D'Urgell (2)
Lisdoonvarna	Ax-les-Thermes
Lisdoonvarna	Carol
Lisdoonvarna	La Seu D'Urgell (2)
Mullaghmore (2)	Carol
Mullaghmore (3)	Carol
Slieve Carran (1)	Ax-les-Thermes
Slieve Carran (1)	Carol
Threecastles	La Seu D'Urgell (2)

Supplementary Table S3 has the complete set of F*_ST_* values.

## Discussion

The majority of *C. nemoralis* individuals from Ireland have a mitochondrial lineage, C, that is in common with Central (Andorran) and Eastern Pyrenean populations. This lineage was found nowhere else in mainland Europe, except one site near Toulouse (St. Sulpice) (Figure 2) and the Isle of Man, as well as previously being found in a single snail from Wales [10]. For a genetic marker that is highly variable, especially within snails [30], it is noteworthy that three shared Irish/Pyrenean haplotypes were found, with no evidence of genetic differentiation between several different sites in Ireland and the Pyrenees (Table 1). Except for the Threecastles location, all of these sites were on the West coast of Ireland, where the white lipped shell morph is most common. In accordance with the pygmy shrew and some other animals [5], the evidence indicates that some Irish *C. nemoralis* have a ‘Lusitanian’ element, previously speculated but never tested [9]. If other Irish species have a similarly cryptic Lusitanian element, then this raises the possibility of a more widespread and significant pattern.

There is an ongoing debate as to the biogeographic history of Ireland, including a recent body of evidence that highlights the importance of Britain in the origin of Irish fauna [6], [31], [32], [33]. In the case of *C. nemoralis* snails, particular caution is required in the interpretation in relation to Britain, because the inferences only reveal the history of the mitochondrial DNA contained within the snails, a single genetic locus, and give no indication for the geographic origin of the rest of the genome [4], [6]. The diverse lineages found within Ireland certainly indicate that the colonisation history is complicated, such that the other lineages may have reached Ireland from similar or different sources, including Britain, at the same or different times (e.g. haplotype D11 is shared between Ireland and Southern France; Ireland A haplotypes are largely distinct from British A haplotypes, though there is some overlap; Supplementary Table S1; Supplementary Figure S1). Here we focus our discussion on the lineage with the clearest pattern, C. for which the simplest explanation for the disjunct distribution is a single historic long distance dispersal event between the Pyrenees and Ireland. ‘Gradualist’ explanations for the distribution of lineage C, involving a stepping stone colonisation from the Pyrenees, through France and into Britain, are not satisfactory, because it is difficult to reconcile how a much slower migration did not result in significant genetic structure, as observed in most other pairwise F*_ST_* comparisons (Figure 3). Moreover, the Pyrenean haplotypes dominate in most populations across Ireland (with the exception of the South-West corner), including near the sites where shells have been radiocarbon dated (Preece *et al.*, 1986; Speller, 2007), indicating that they might be more common because they arrived first.

One significant possibility is that lineage C might exist in other countries, but was not sampled, or else it has since gone extinct [34], perhaps due to climate induced replacement [4]. Even so, the lack of structure between Ireland and the Pyrenees is difficult to explain by anything but long distance migration, and, whichever the precise explanation, the differentiation between Ireland and Britain is consistent with the model put forward [4], [6] in which Ireland may retain a signal of founding colonization for longer than Britain, simply because subsequent waves of colonisation did not take place.

In Ireland, anthropogenic disturbance implies that people colonised Ireland at least 9000 years B.P., more or less coincident with the earliest post-glacial *C. nemoralis* fossils (8207+/−165, Co. Mayo). Molecular genetic evidence indicates this resettlement of much of western and central Europe originated from the Franco-Cantabrian or Iberian region [35], [36]. Subsequently, trading links were established between Iberia and Ireland in the Mesolithic, providing ample opportunities for land snails to be transported in the cargo [37], [38]. Intriguingly, Pyrenean and Cantabrian humans have consumed *C. nemoralis* since at least the end of the Pleistocene (e.g. shells radiocarbon dated from 10,932±196 cal BP) [39] and they are commonly found as an item of food refuse in Mediterranean archaeological sites just prior to the onset of agricultural economies (though not in Ireland – Eva Laurie, pers. comm.). Specifically, deep middens of burnt shells have been found during Pyrenean cave excavations, indicating that humans have either been collecting or possibly “farming” land snails for thousands of years, with the majority of these shells being *C. nemoralis* [37], [40]. Archaeological evidence from a Bronze Age ship wreck in Turkey also indicates that people have transported land snails, either intentionally or accidentally, across oceans for at least 3000 years [41].

Another possibility is that individual snails could have also reached Ireland via other means, most likely rafting on floating debris in the ocean or accidental transport by birds. However, rafting seems unlikely, given that the C lineage is found in the Central Pyrenees and, so far, not on the Atlantic coast of Spain or France. Transport by birds is possible, but we are not aware of any large bird species that migrate between Ireland and the Pyrenees [42] and also, *Cepaea* does not have particularly ‘sticky’ mucus [43], although ingestion remains a possibility [44]. Finally, natural colonization after the last glacial maximum over land now covered by sea cannot be ruled out [3], but this pattern of colonization would in theory leave a stepping stone signature of population structure (unless very rapid) [45], at the same time as leaving remnant haplotypes of the same mitochondrial lineage in places such as the West coast of France – these may exist but we did not discover them.

It has been carefully argued by Cunliffe [38] that the maritime communities of the Atlantic, and the rivers that drain into it, were well connected along the coast, but less so inland. Taking the evidence together, we therefore believe that while it is not possible to rule out other explanations, the most likely justification for the specific connection between Irish and Pyrenean *C. nemoralis* is human assisted movement of snails, a mode of transport that has been inferred for some other species [5], [46]. While the DNA evidence from these snails may therefore reveal an insight into the journeys taken by early humans, an intriguing question is why the Irish C lineage haplotype appears to have originated from an area that is approximately 100 km inland? In the present day, *C. nemoralis* is not found much further East than our sample sites, and certainly not on the coast near Perpignan and Girona. One explanation is that the Garonne river, into which the Ariège on the northern flanks of the Pyrenees drains, has long been a principal route between the Mediterranean and the Atlantic [38], perhaps implying that the present day presence of these snails in Ireland might be a long lasting consequence of this ancient transport route. Although beyond the scope of this study, further study would benefit from corroborating these results using recently developed nuclear markers [47], as well as detailed sampling along the course of the Garonne. Whether the same ‘Garonne trade’ explanation may apply to explain the distribution of other species may only be determined by further genetic work.

## Supporting Information

Figure S1 **Median-joining networks showing the relationships between haplotypes within different lineages, with the size of the circle being proportional to the number sampled.** Dashes indicate hypothesised but unsampled haplotypes. Shading represents the location of individual haplotypes, black for Ireland and the Isle of Man, grey for Spain and France, white for Britain, stippled for other locations.
(PDF)

Table S1 **Measures of mitochondrial cytochrome oxidase subunit I genetic diversity and neutrality test statistics for samples new to this study.**
(XLSX)

Table S2 **Mitochondrial 16S rRNA haplotypes found at each site.**
(XLSX)

Table S3 **Estimates of pairwise population structure between sites.**
(XLSX)
