# Supplementary figures and images for: Irish Cepaea nemoralis Land Snails Have a Cryptic Franco-Iberian Origin That Is Most Easily Explained by the Movements of Mesolithic Humans
Source: PLoS One. 2013 Jun 19;8(6):e65792. doi: 10.1371/journal.pone.0065792 (PMC3686809; doi:10.1371/journal.pone.0065792)

Figure S1 a)  
Lineage A

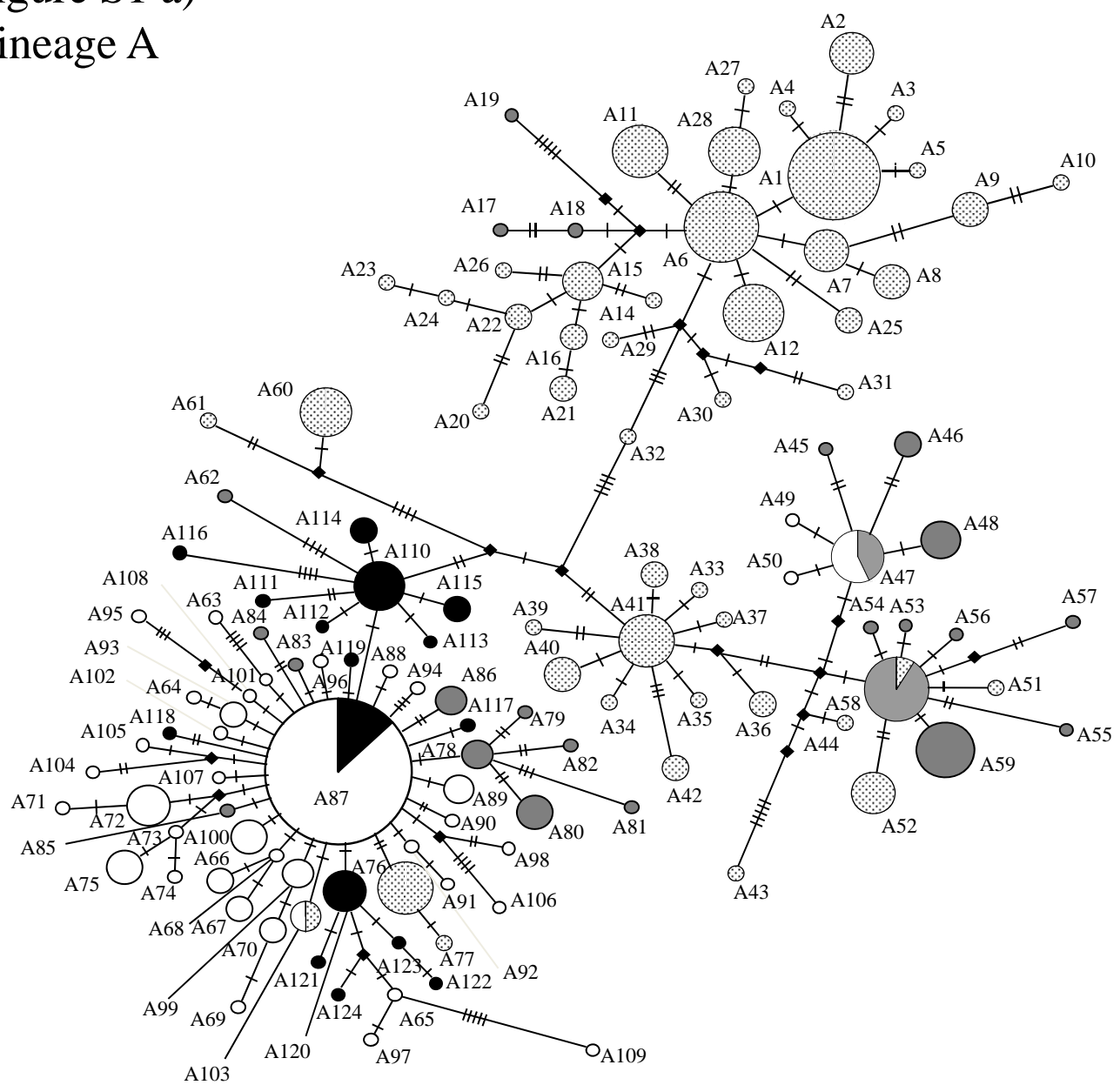

Figure S1 b)  
Lineage B

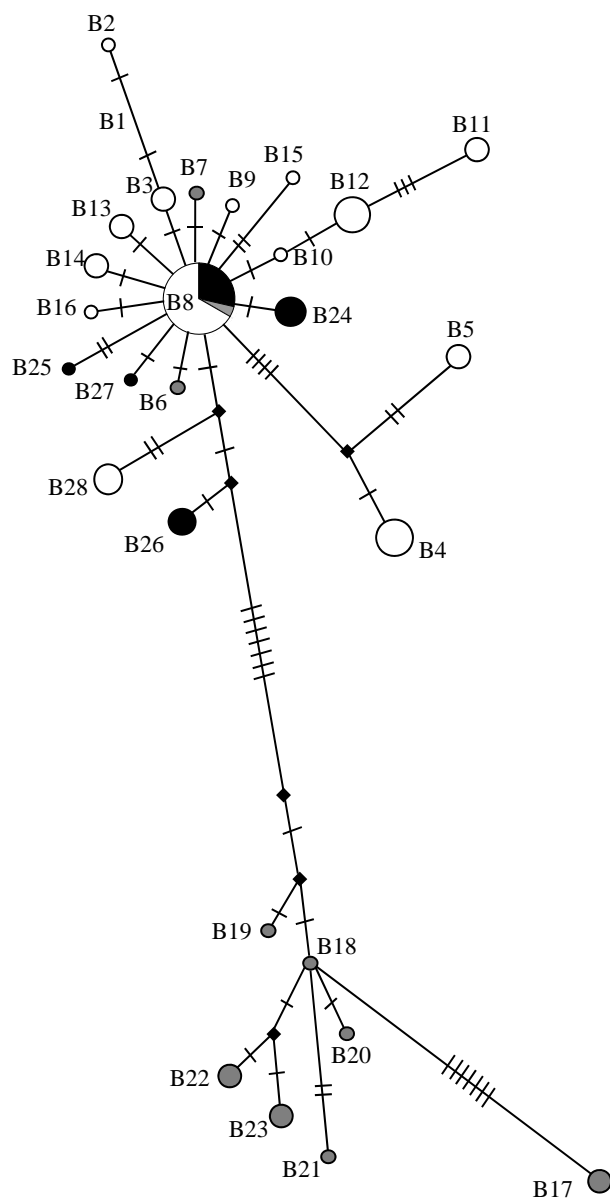

## Lineage C

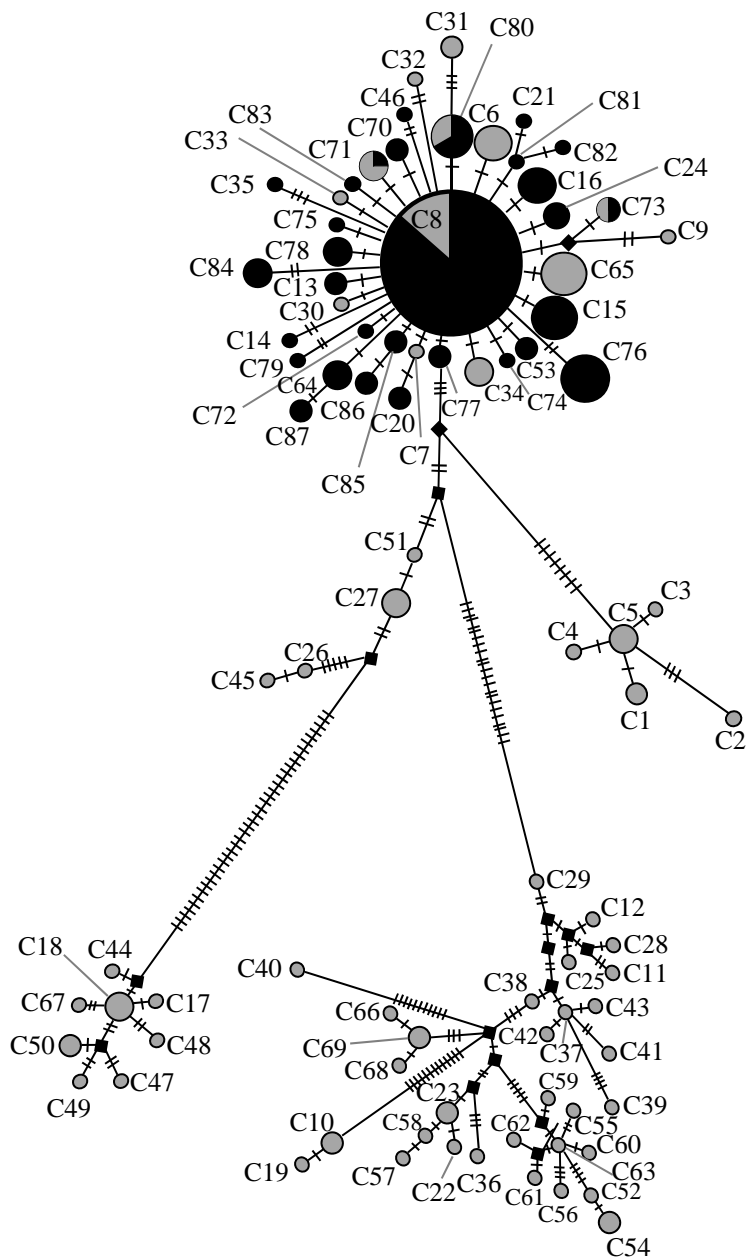

Figure S1 d)  
Lineage D

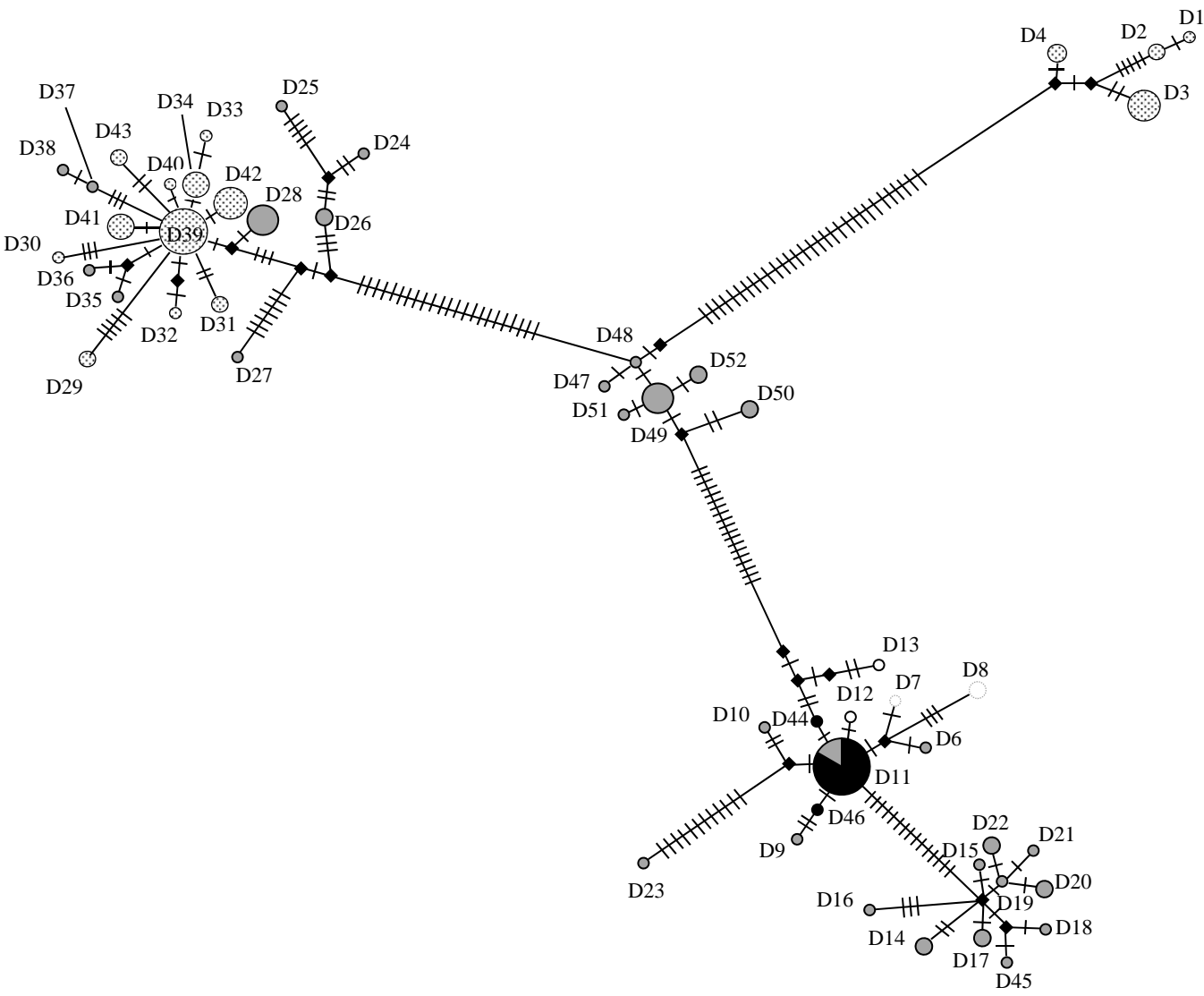

Figure S1 e)  
Lineage F

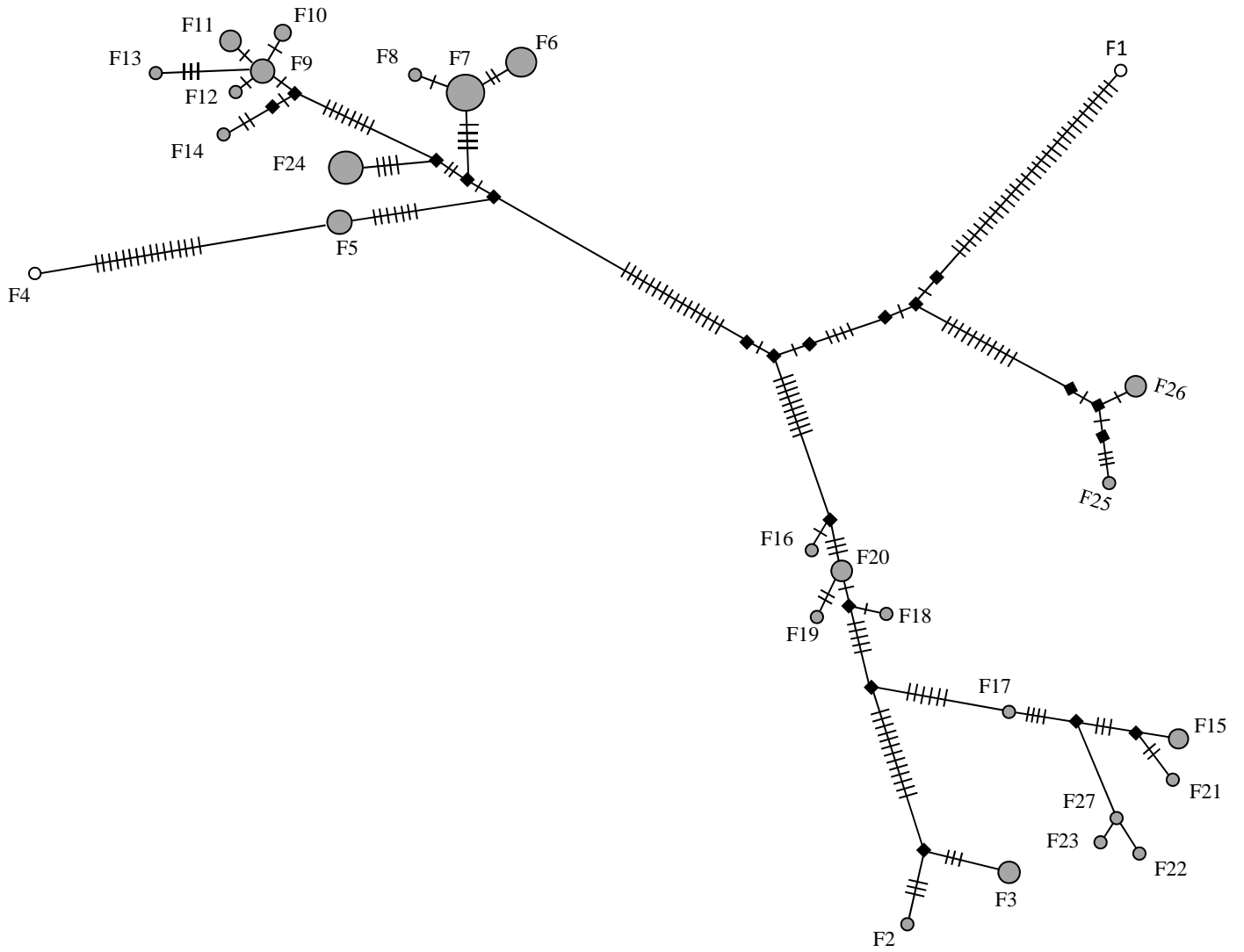

Supplement: Figure S1 — Median-joining networks showing the relationships between haplotypes within different lineages, with the size of the circle being proportional to the number sampled. Dashes indicate hypothesised but unsampled haplotypes. Shading represents the location of individual haplotypes, black for Ireland and the Isle of Man, grey for Spain and France, white for Britain, stippled for other locations. (PDF) [file pone.0065792.s001.pdf]
